# Structural controls on gold deposits in the Egyptian Nubian Shield: insights from the Atalla-Sid Shear Belt, Central Eastern Desert, Egypt

**DOI:** 10.1038/s41598-026-60088-7

**Published:** 2026-08-25

**Authors:** Zakaria Hamimi, Mohamed Abd El Monsef, Hassan El Sundoly, Ashraf S. Abdelmaksoud, Mahmoud K. Alawy

**Affiliations:** 1 Geology Department, Faculty of Science, Benha University, Benha, 13518 Egypt; 2 Petroleum & Mining Geology program, Galala University, Suez Governorate, 435611 Egypt; 3 Geology Department, Faculty of Science, Tanta University, Tanta, 31527 Egypt; 4 Nuclear Materials Authority, 530 El-Maadi, Cairo, Egypt; 5 Geology Department, Faculty of Science, Menofia University, Shiben El Kom, 51123 Egypt; 6 Geology Department, Faculty of Science, Alexandria University, Alexandria, 21511 Egypt

**Keywords:** Egyptian NubianShield, East African Orogen, Orogenic gold, Atalla-Sid Shear Belt, Paleostress analysis, Planetary science, Solid Earth sciences

## Abstract

Within the Egyptian Nubian Shield (ENS), the majority of gold deposits are orogenic, occur in a wide range of tectonic settings and commonly exhibit distinct spatial and temporal distributions. Gold mineralization is predominantly hosted within quartz and/or carbonate veins and veinlets emplaced along late Neoproterozoic structural features, including megashears, fractures, stockwork systems, brecciated and mylonitized zones, hinges of thrust- and shear-related folds, and tectonic contacts. The Atalla-Sid Shear Belt (ASSB) is one of the eye-catching gold-bearing megashears in the ENS encompassing two historic gold mines (Atalla and Sid). In this work, detailed field/structural investigation was carried out and integrated with paleostress and fracture analysis to establish a tectonic model and to provide a template for the ENS analogous terranes. Four paleostress tensor stages are indicated in this zone. Stage 1 involves transpressional regime which with a prolate shape of the stress ellipsoid (R′= 1.5) and NNW-SSE SH_max_ (SH_max_ = N153﻿°E) coinciding with the Najd Shear orogeny. Stage 2 marks a switch to transtensional regime with a oblate shape of the stress ellipsoid (R′= 1.5) and ENE-WSW SH_max_ (SH_max_ = N074E). Stage 2 played a significant role in controlling gold distribution. The transition between both stages is likely triggered by crustal relaxation and a change in the far-field stress orientation. Stage 3 represents extensional regime (R′= 0.5) and ENE-WSW Sh_min_ (Sh_min_ = N064﻿°E); and Stage 4 indicated a compression regime (R′= 2.5) and NE-WSW SH_max_ (SH_max_ = N039﻿°E). Stages 3 and 4 represent the final phases of the Neoproterozoic Orogeny that played a role in the final structural geometry of the belt without a noticeable role in gold mineralization. Results obtained from the analysis of fault pattern data and processing of photographic fracture networks demonstrate three-abundance orientations (NW, NE and E) of fracture population in Atalla area, and two orientations (NW and NE) in Sid area. It is recommended to consider stage 2-related shearing in further exploration programs in the ENS.

## Introduction

Gold mining started ~ 6000 years ago in Egypt. From Egyptians, other civilizations (e.g. Greeks, Persians and Romans) learned the techniques of gold exploration, mining and purification^1^. By the mid nineteenth century, two main auriferous deposits have been known; lode (vein) deposits and placers, and the origin of vein-type gold deposits has been interpreted within the frame of four main theories or hypotheses prevailing at that time; pure magmatic, magmatic-hydrothermal-, secretion- and abyssal-theories. The auriferous veins occur in a wide range of litho-units and receive their gold by various mechanisms from numerous sources. Vein-type to disseminated gold deposits is generally classified as orogenic gold deposits (OGDs). Boyle^1^ enumerates several types other of auriferous deposits and one them is veins and saddle reefs in fractures, folds, crushed zones and bedding-plane discontinuities.

In the last decades, gold deposits in the Egyptian Nubian Sheild (northwestern Arabian-Nubian Shield, ANS, and East African Orogen, EAO) have been extensively studied. Most of these studies focused on genesis of gold and general structural controls on gold mineralization. There were rare of few studies that focused on detailed fracture patterns that host gold deposits. Gold deposits in the Central Eastern Desert exhibit a significant spatial relationship with ophiolites and prominent fault structures, as identified by Zoheir and Moritz^2^. In certain granite-hosted deposits, mineralization is commonly limited to shear zones formed after an intrusion, this could indicate that the physical properties of the solid granite rocks influenced the creation of the structural framework^3^.

**Fig. 1:**
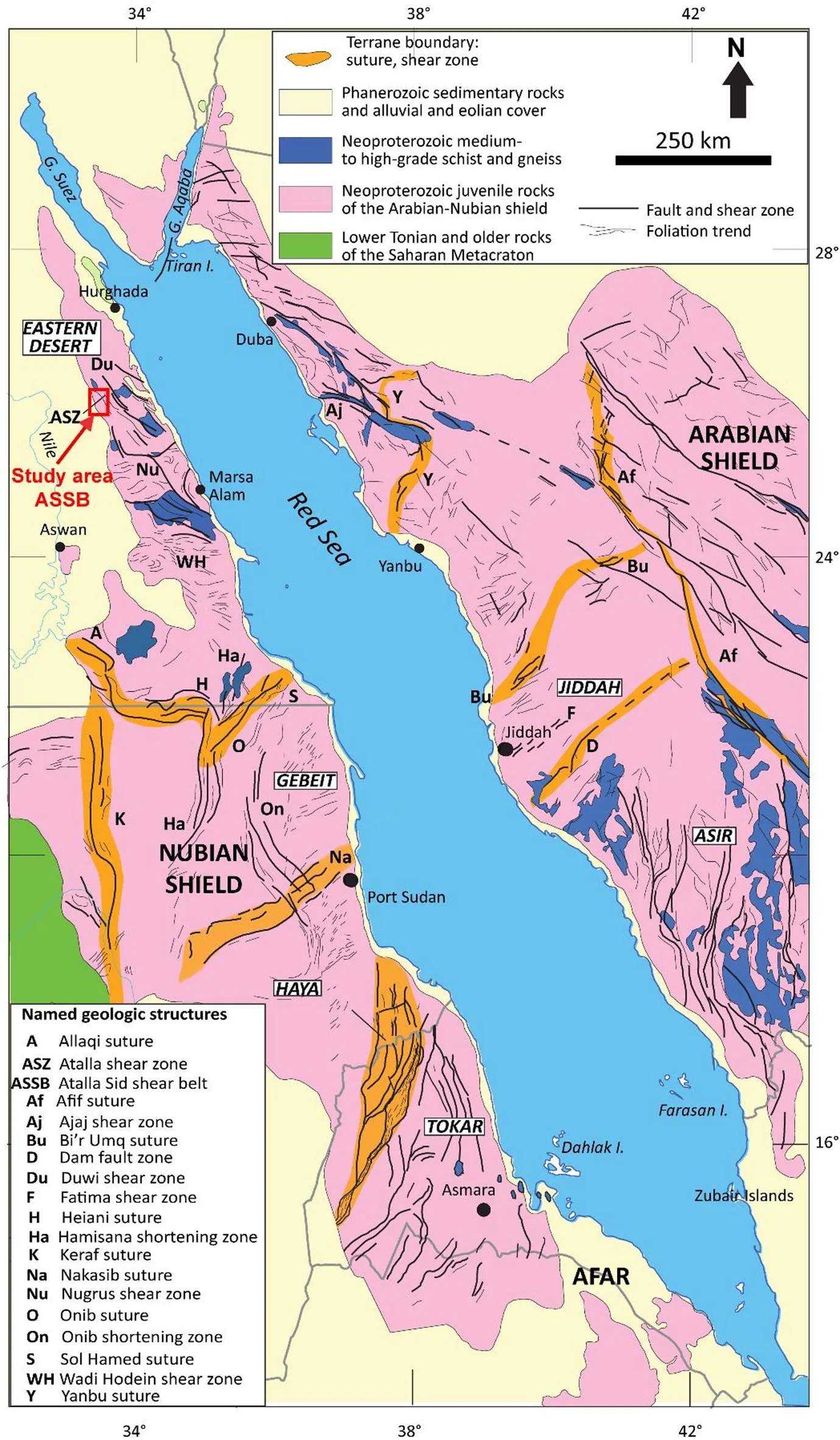
Location map of the study area (Red box) within the uplifted margins of the Arabian and Nubian Shield (ANS; modified after^10^).

The Atalla-Sid Shear Belt (ASSB) area has attracted significant interest because of its advantageous position along the Qift–Quseir road, Central Eastern Desert, Egypt. This region serves as a prime example of the Egyptian Basement Succession found in the Eastern Desert (ED), leading to numerous investigations involving petrology, geochemistry^4–6^ and structural analysis^7–9^. The area in the CED of Egypt spans 100 square kilometers, bounded by latitudes 26° 0′ to 26°10′ N and longitudes 33° 28′ to 33°48′ E (Fig. 1). It is characterized by notable topographical landmarks like the Meatiq Gneiss Dome, Gabal Um Had, Gabal El Rubbshi, and renowned valleys (Wades), such as Wadi Atalla, Wadi Um Had, and Wadi Hammamat. Fowler^11^ proposed that the presence of structural discontinuities such as domed shear zones and moderately dipping thrusts in the CED aided in the emplacement of calc-alkaline granitoid intrusions, several of which are linked to hydrothermal processes and the formation of gold deposits.

Paleostress analysis used to reconstruct the principal stress orientations and tectonic regime that controlled deformation in the younger granite, aiding in identifying the time and conditions of fractures formations. On the other hand, fracture analysis characterizes the distribution, orientation, conductivity, and permeability of fractures which act as pathways for hydrothermal fluids. By integrating both approaches, it becomes possible to determine which fractures were favorably oriented and open during fluid flow, thus controlling gold mineralization.

**Fig. 2:**
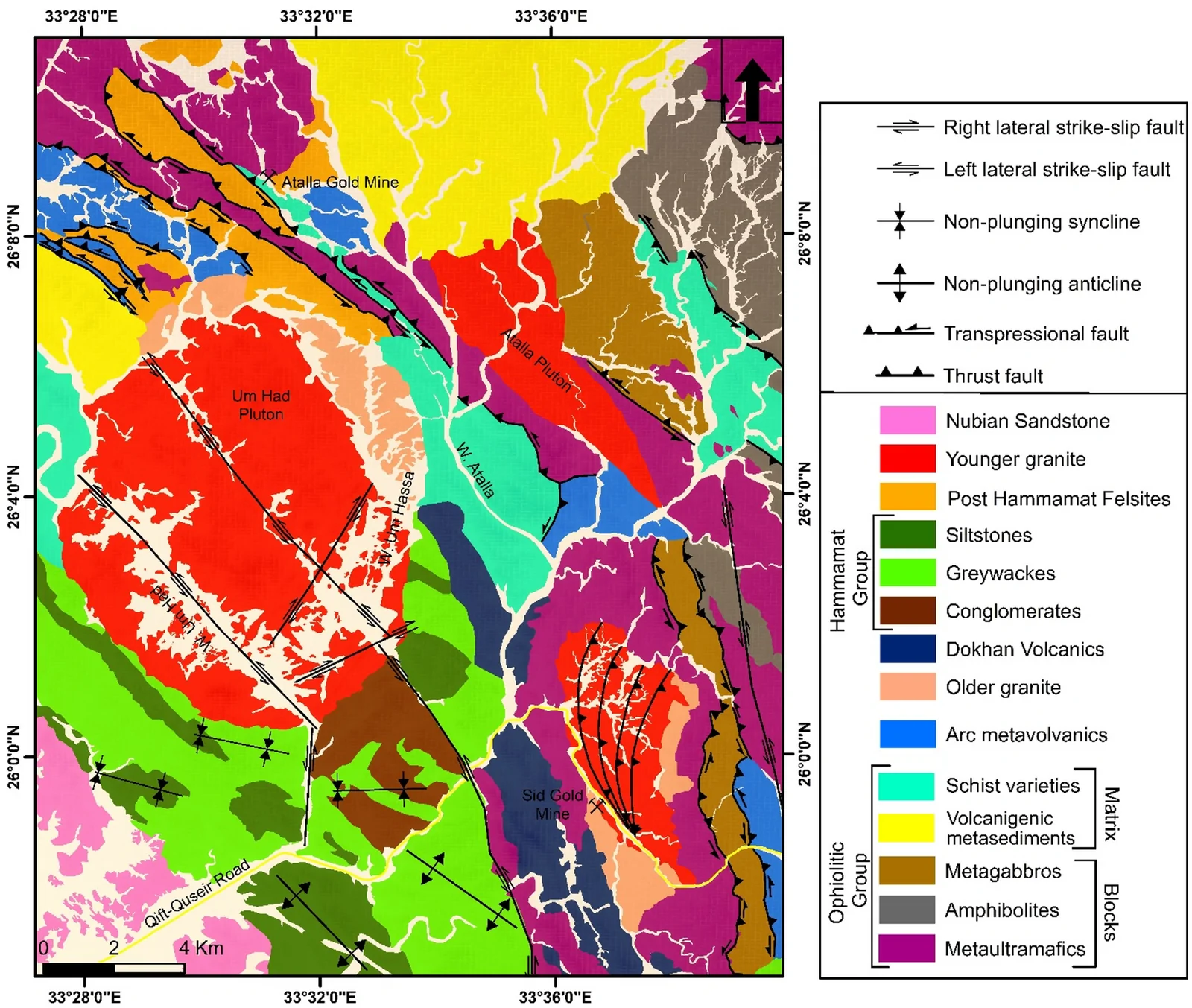
Geologic map for Atalla Sid Shear Belt area (ASSB, modified after^12^).

In the present work, detailed fault straie analysis and paleostress reconstruction, together with the analysis of fault pattern data and processing of photographic fracture networks, have been carried out for the first time on the study area. Results obtained from the current study could significantly contribute to understanding the main role of structural elements in controlling gold mineralization, by identifying the deformation stage associated with mineralization concentration. This will aid gold exploration in other areas all over the shield. In the ASSB, two historic gold mines, namely Atalla and Sid^12^ (Fig. 2), are well-known and shear zones seem to have controlled vein-forming fluid flow. In Addition, both Atalla and Sid areas frequently show expectable and monotonous structural geometries. Our study aims to decipher the structural control mechanisms on gold mineralization within the ASSB which represents a key shear belt, in the ANS. The results obtained provide further insights into the role of structural discontinuities in the formation of the OGDs. Ultimately, the findings will enhance our understanding of tectonic evolution in the ANS and provide a scientific basis for identifying new gold exploration targets.

## Geological background

### Regional geological framework

The ANS belt is located to the north that narrows down southward and serves as mega suture zone between east and west Gondwana at the northern extremity of the EAO^13–21^ (Fig. 1). Stern and Hedge^22^ classified the Eastern Desert of Egypt into three main regions: the Northern, Central, and South-Eastern Deserts (NED, CED, and SED). Fowler and Osman^23^ underscored the importance of this separation within the larger tectonic examination of the ANS and EAO. The provinces are situated side by side, separated by two key structural features. The Qena-Safaga shear zone serves as a boundary between the NED and CED provinces, while the Idfu-Mersa Alam shear zone divides the CED and SED provinces. The Basement rocks of Egypt constitute part of the ANS which is called ENS. Recently, Hamimi et al.^24^constructed the first tectonic map for the ENS and discriminated three tectonic provinces, which are: North Extensional Province (NEP), Central Wrench Province (CWP), and South Compressional Province (SCP). These provinces evolved through three major kinematic events: (1) N-S convergence (c. 800–620 Ma) along the YOSHGAH suture, (2) the Nabitah Orogeny (c. 640–580 Ma) involving terrane accretion, and (3) the retreat of the Cadomian Arc (younger than c. 580 Ma). The dominant structural fabric elements in the SCP are the imbricated thrust stacks and WNW-ESE to W-E oriented thrusting, both of which are prominently located in the vicinity of Wadi Allaqi’s northern flank^24^. Thrust propagation folds created as a result of the S- (to SSW-) propagated thrusts can be traced throughout this province, extending as far north as Wadi El Gemal. Going to the north, the effect of Najd Shear Corridor is well noticed, where it overprinted the previous structures. In addition, many studies^20,25–33^ related the exhumation of the gneissic domes or metamorphic core complexes in this province (Fig. 1) (e.g. Hafafit, Meatiq, and Sibai) to transpressional shear zones. The northern extensional province is dominated by extensional structures due to extension in N-S direction. These structures are manifested by the voluminous granitoids (e.g. Gabal Gattar), the E–W trending dyke swarms^24^.

### Geology of the Atalla-Sid Shear Belt (ASSB)

**Fig. 3:**
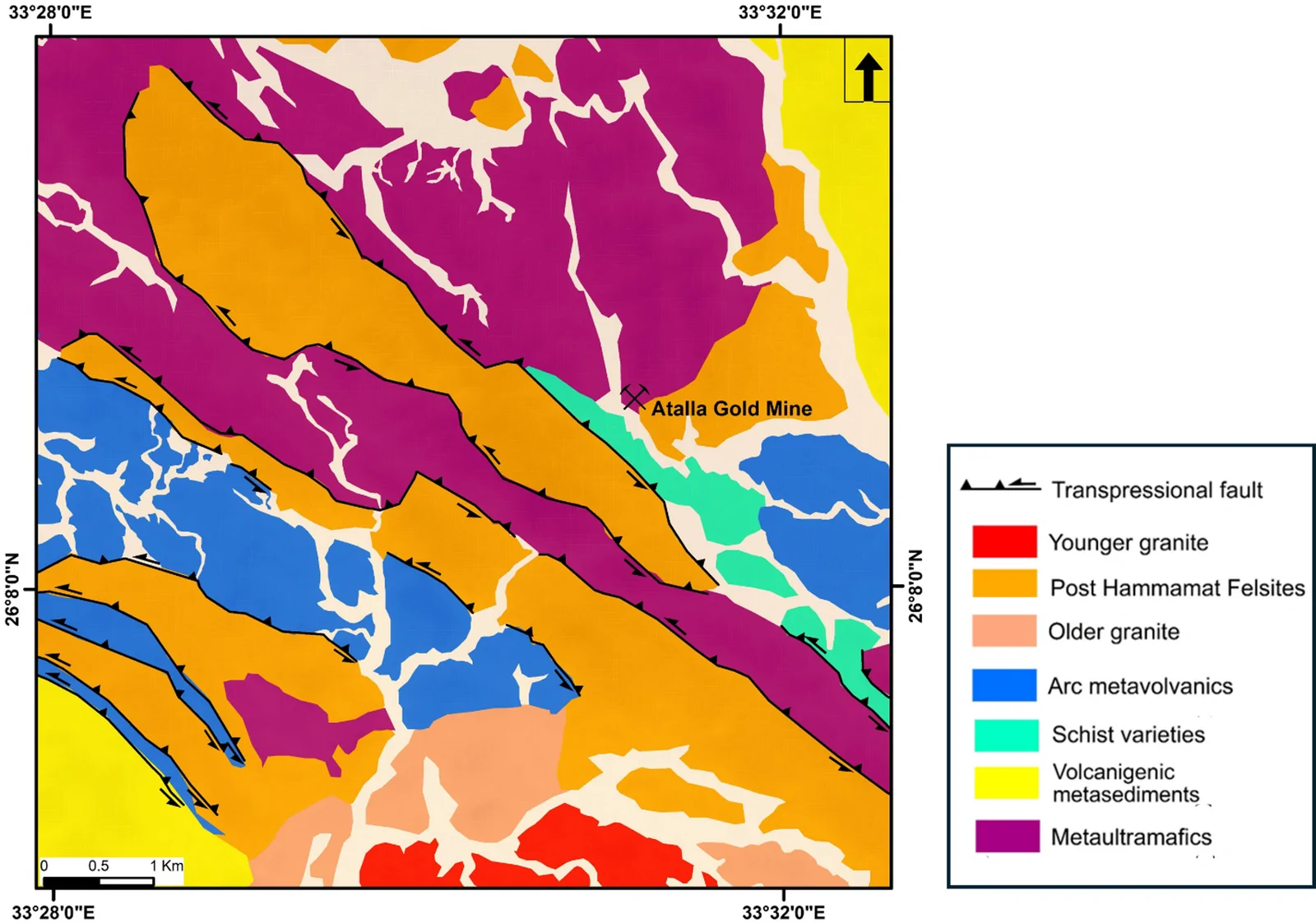
Geologic map for Atalla area including Atalla Gold Mine (AGM, modified^12^). GPS coordinates: 26° 8’56.00"N, 33°31’12.00"E.

**Fig. 4:**
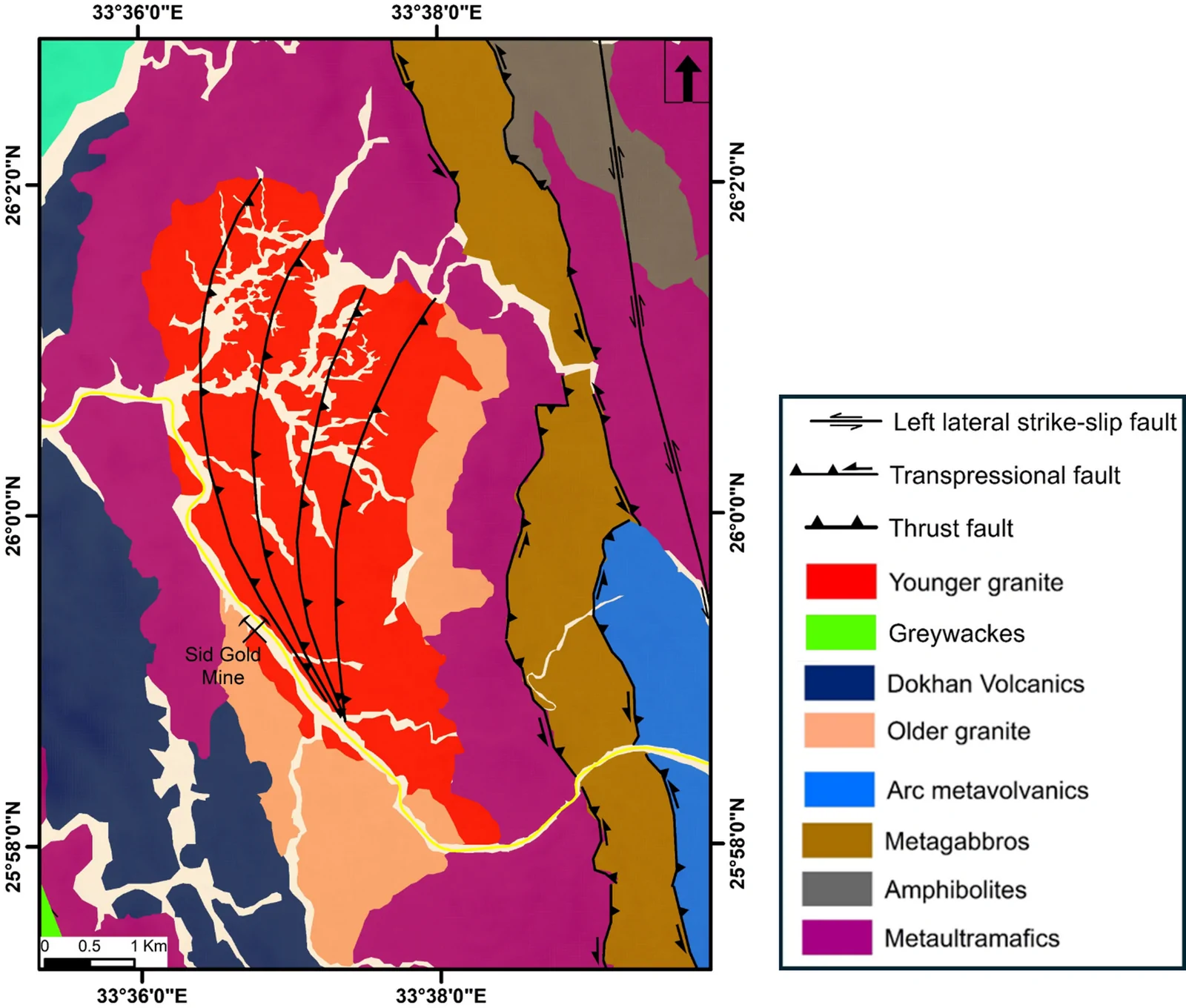
Geologic map for Sid area including Sid Gold Mine (SGM, modified after^12^). GPS coordinates: 25°59’19.19"N, 33°36’46.68"E.

Integration of the present detailed field work, high resolution Google Earth images of up to 1 m/pixel resolution, in addition to the previous mapping and age determinations were used to construct the present maps for the study area (Figs. 2, 3 and 4). The rock succession includes Neoproterozoic rocks that were represented by ophiolitic mélange association, island arc assemblage, Dokhan volcanics and Hammamat molasse sediments (Fig. 2). These rocks were intruded by syn-tectonic granodiorite, post-collision younger granites (615–570 Ma^34^), post Hammamat felsites^35–39^and different types of dykes and quartz veins. The Atalla-Sid ophiolitic sequence occurs along Qift-Quseir road, about 80 km from Quseir^40–43^. Specifically, it is dispersed around Sid Gold Mine and extended around Wadi Atalla till reaching Atalla Gold Mine. The whole sequence was intruded by the post-orogenic Fawakheir younger granite pluton. The serpentinites are serpentinized peridotite and/or dunite that are transformed to talc carbonate, siliceous rocks, and quartz-carbonates (listwanite) bodies along Atalla shear zones. The metagabbros are coarse-grained and grade into fine-grained metagabbros. The basic metavolcanics comprise massive metavolcanics and pillow lavas, which are locally brecciated. The pillow lavas are commonly spheroidal with chilled margin. The sheeted dikes were oriented in a NW–SE direction and dip mostly to the NE.

Arc metavolcanics are represented mainly in the southeast and northwest of the entire area. Minor intrusions are exposed to the east of Atalla Gold Mine and to the south of Atalla granitic pluton (Fig. 2). Older granite (syn-tectonic granite) exposed to the south and east of Fawakheir younger granite pluton and to the north of Um Had pluton (Um Sheqila). In Um Sheqila, it is represented by coarse-grained granodiorite to tonalite in composition^12^.

Dokhan volcanics are located to the east-southeast of Um Had pluton and to the west of Fawakheir ophiolitic sequence^44,45^. They extend as a long belt with the same trend of ASZ and are characterized by a buff to black color and a fine-grained texture. Hammamat Group Sediments are exposed around wadi Hammamat to the south of Um Had pluton where the best exposure was found in the Central Eastern Desert (type locality)^37^^,39^. Post Hammamat felsites were recorded in many locations in the Eastern Desert^35,38,46^. In the concerned belt, they outcrop as NW-SE narrow belt to the NW of Atalla Gold Mine (Figs. 2 and 3). They mainly consist of rhyolites and intrude metaultramafics, metavolcanics, and Um Sheqila granodiorite. Younger granites are represented by three main plutons which are Um Had, Fawakheir, and Atalla plutons (Fig. 2). Um Had is roughly a circular pluton with monzogranite-synogranite composition^3^ and intruded Hammamat sediments where the contact is sharp (Fig. 2). Fawakheir and Atalla plutons consist of alkali feldspar granite^47^ with roughly oval shape and intrude the ophiolitic sequence.

### Structural elements in the Atalla-Sid Shear Belt

**Fig. 5:**
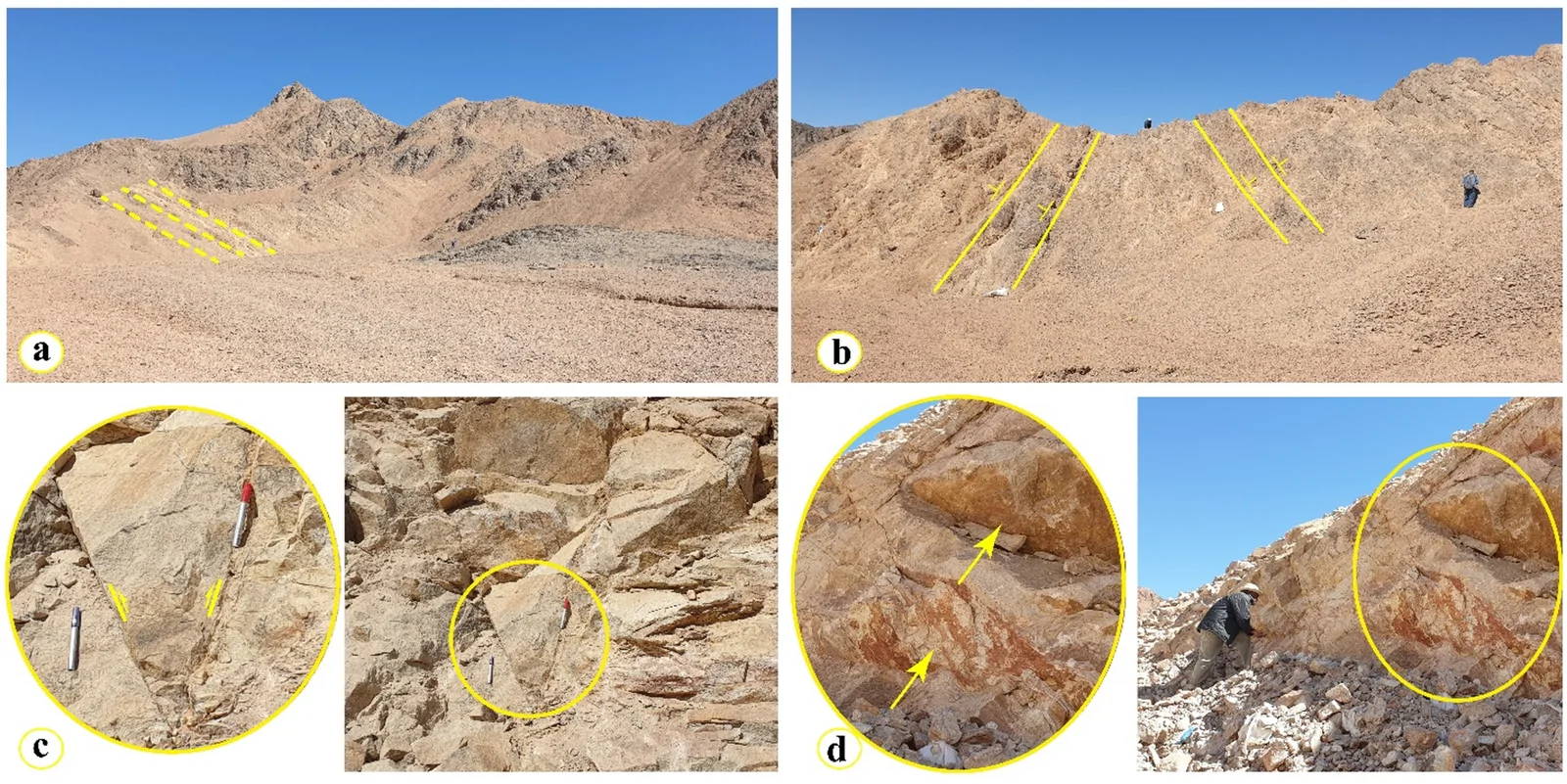
Field photographs of some outcrops in Atalla area showing (**a**) the main shearing trend, looking NE (**b**) imbricated thrust stacking, looking NE, (**c**) conjugate fracturing, top view, and (**d**) alteration along the main shearing planes, looking SW.

**Fig. 6:**
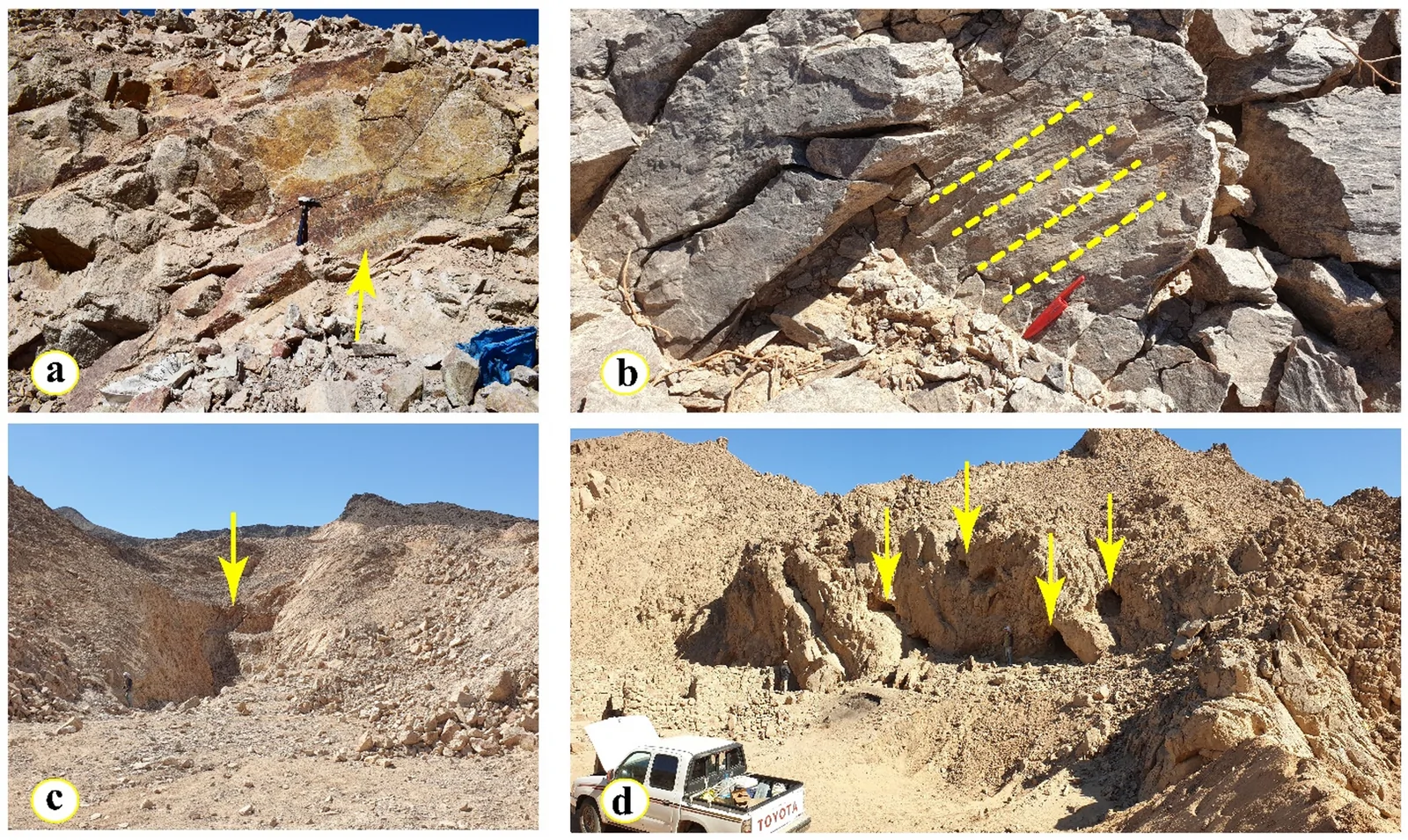
(**a**) The main shearing planes, looking NW, (**b**) Oblique slip lineation in Atalla area, top view (**c**) and (**d**) illegally dug trenches by the “Dahhaba”, (**c**) looking SW, (**d**) looking NE.

**Fig. 7:**
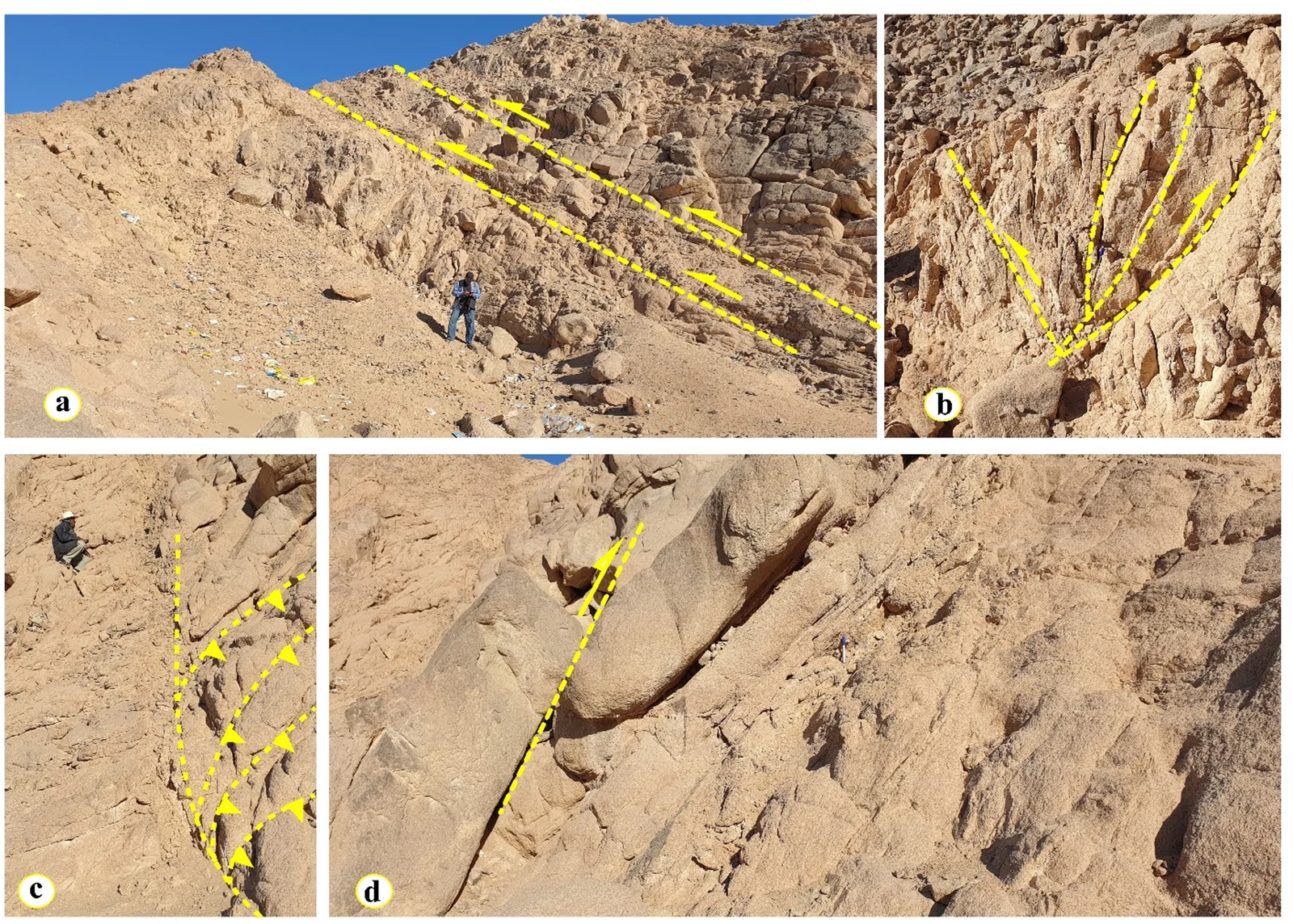
Field photographs of some structural features in Sid area showing (**a**) imbricated thrust sheets, looking NE (**b**) positive flower structure, looking NE (**c**) wrench-induced thrusting, looking NE (**d**) obvious thrusting, looking NE.

**Fig. 8:**
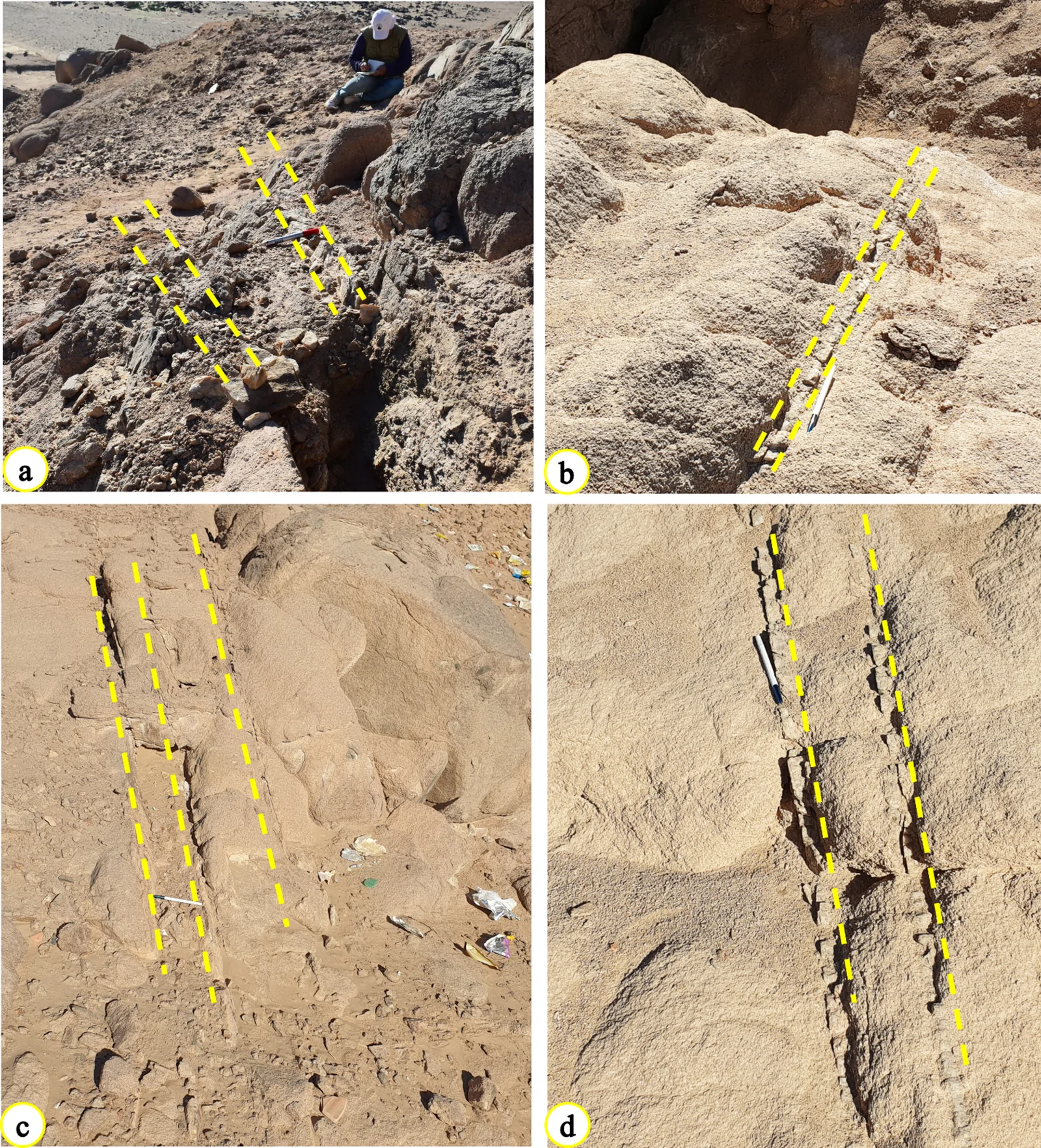
(**a-d**) Gold bearing quartz veins injected along N60º−70ºE transtensional direction, (**a**) looking SW, (**b-d**) looking NE.

The area of study lies in and shows most of the essential structural features of the Central Wrench Provence (CWP). A wide variety of geologic structures such as thrusting and thrust-related structures, folds, shearing and shear zone-related structures, strike-slip faults, foliations, and lineations can be observed. These structures range in size from microscopic or even sub-microscopic scale up to mountainous scale. NW-SE trending thrust faults are a widespread structural element with NE dipping thrusts and SW-ward tectonic transport. They mark the tectonic boundaries between metaultramafics, amphibolites, metagabbro blocks, mélange matrix and the arc assemblage in Sid area **(**Figs. 2 and 4). These thrusts and associated NW-SE trending folds have been considered to be formed due to shortening in ESE- to ENE elsewhere in the CED^30,48–51^. Furthermore, a younger set of thrusts has been noticed in Fawakheir granitic pluton and post-dating the granitic intrusion^12^ (Figs. 2 and 4). Our field investigations indicated intensive degree of shearing and mylonitization along thrust planes (Fig. 5a), local thrust sheets between ophiolitic blocks, and thrust related folds (Fig. 5b). Fritz et al.^25^ proposed a wrench corridor model in which oblique convergence is partitioned into ENE-WSW compression and NW-SE sinistral strike-slips shears zones (Najd Fault System). This wrench model has been used to explain the dominant NW-SE structural framework prevalent in the eastern desert of Egypt. ASSB has been documented as one of the well-defined NW-SE transpressional sinistral strike-slip shear zone in the CED^12,^^25,^^50^^,52^. Besides, compressional related structures, pervasive NW-SE subvertical foliations (Fig. 6a) and NW and subhorizontal lineations have been noticed through field work as evidence for dominating NW-SE strike-slip movements post-dating ENE-WSW compression. Oblique slip lineations along these fault planes have been traced during field work indicating components of sinistral strike-slip movements (Fig. 6b). ENE- to E-W dextral shear trends have been noticed transecting the whole rock successions through the belt. The ideas about kinematic history of this trend is controversial. El Gaby et al.^53,54^ considered this shear trend and the NW-SE one as a conjugate pair, whereas Akawy^12,^^55^^,56^, considered it as the youngest (extensional) deformation phase. This phase is evidenced by many extensional fractures with different sets and filled quartz veins (Fig. 5c, d). It is worth mentioning that other obvious NE-, ENE-, N- to NNE shear zones, such as Qena-Safaga, Idfu-Mersa Alam, and Hamisana have been documented through the eastern desert^57^. Furthermore, many unregulated mining tunnels in NE-SW directions in Atalla Gold Mine (Figs. 6c) and drilling holes in ENE-WSE directions in Sid Gold Mine have been observed during field work (Fig. 6d). Also, we delineated many evidences for dextral ENE-WSW shear trend in many locations, especially in Sid Gold Mine area. Besides, imbricated thrust sheets (Fig. 7a), fault planes with striations and different kinds of structures (e.g., positive flower structure and wrench-induced thrusting, Figs. 7b, c,d) are also observed. Moreover, numerous gold-bearing quartz veins with N60°−70°E direction (Fig. 8) have been traced, especially around drilling holes and tunnels. Hammamat molasse basin, which lies to the southwest of the concerned shear belt, has also preserved different major structural elements such as NW-SE trending sinistral (Najd) shear zones, ENE-WSW dextral shear zones, E-W to NE-SW trending folds and thrusts (Fig. 2); NW-SE trending folds and thrusts like most of molasse basins in the CED^12,58–62^.

## Methodology

### Paleostress data acquisition and processing

In order to identify the dominate tectonic stress during faulting and analyze the fault kinematics, fault-slip data have been studied in two different subset areas. Following strain or stress path to interpret fault-slip data is a long controversial issue^62-^^64^. Utilizing kinematic methodology only, strain or strain rate can preferably be resolve than stress. This method drawback is that we lack information about the displacement amount on each measured fault. However, Fossen^65^ stated that considering a series of hypotheses fault slip data can be used to reconstruct paleostress tensor. These assumptions include uniform stress field in the study’s purview, no discontinuities (e.g. mechanical layering) or interaction between faults, and the stress field responsible for formation of fault population should be constant and single during the history of faulting. We bear in mind that these requirements are not always met and also careful interpretation of paleostress results should be done taking in consideration the local characteristics. However, a wealth of experience with paleostress inversion has shown that it is a useful tool for reconstructing stress fields and interpreting tectonics^64^^,66^.

In this study, we employed the traditional method that relies on the rock mechanics for paleostress reconstruction^62,67–69^. This method is based on the fact that there are four parameters of stress tensor used to determine the faulting geometries. These parameters are the orientation of the three principal stress axes (σ1, σ2, σ3) representing the maximum, intermediate, and minimum compressive stresses, respectively, and the ratio of their relative magnitude R (σ2−σ3/σ1−σ3). Conversely, these four parameters that make up the reduced paleostress tensor can be reconstructed using differently oriented set of faults and fractures.

Win_Tensor program^70-^^72^ in association with the methodology described in^73^ and used in many case studies^74^^,^^75^ have been used in dealing with fault-slip data in the present study. In this program, the rotational optimization iterative method was utilized in conjunction with the F5 function that minimizes the angular deviation between the noticed and resolved slip direction simultaneously and promotes slip on fault planes. The reduced stress tensor’s four parameters are additionally reduced to a combination of the horizontal stress axes SHmax and Shmin^76^, and the stress regime index R′^74^. To display the R-values for different tectonic regimes, the parameter R′ is a very effective way where R′=2 + R for compressive regimes (σ3 is vertical), R′=R for extensive regimes when σ1 is vertical, and R′=2-R for strike-slip regimes (σ2 is vertical). The terms “Shmin” and “SHmax”, which are orthogonal to one another, denote the lowest and maximum horizontal principal stresses, respectively.

We have divided the data into subsets based on our knowledge from field observations that the fault data belongs to distinct subsets, and we performed a separate stress inversion for each subset. The subsets were defined chiefly based on field-based cross-cutting or overprinting relations observed at the outcrop scale. The manual separation was preferred over automated clustering to ensure that each subset is strictly constrained by field-observed kinematic indicators and relative age relations. Stress inversion was performed in a continual manner using the initial stress tensor as a guide to draw compatible data. The stress tensor is differentiated based on the orientation of the stress axes, and stress ratio R. We performed a cross-check to confirm that the fault-data had been assigned to the correct subset after the final determination of the fault subset and their stress tensor. This has been done by applying the relevant stress tensor to the data and determining the resolved normal and shear stress magnitudes and orientations.

### Fault pattern data analysis

The patterns of fractures significantly affect rocks’ mechanical and transport characteristics^77^. Studying and recognizing fracture patterns is influential in numerous earth science fields, such as structural geology, tectonics, and hydrogeology, depending on the geometrical characteristics of the individual cracks and their collective ensemble^78-^^81^. Understanding the physics behind an observable fracture pattern is a prerequisite for making reliable predictions about its extent and scaling in the subsurface, which ultimately discovers the transport characteristics of the network (i.e., permeability)^77^.

**Fig. 9:**
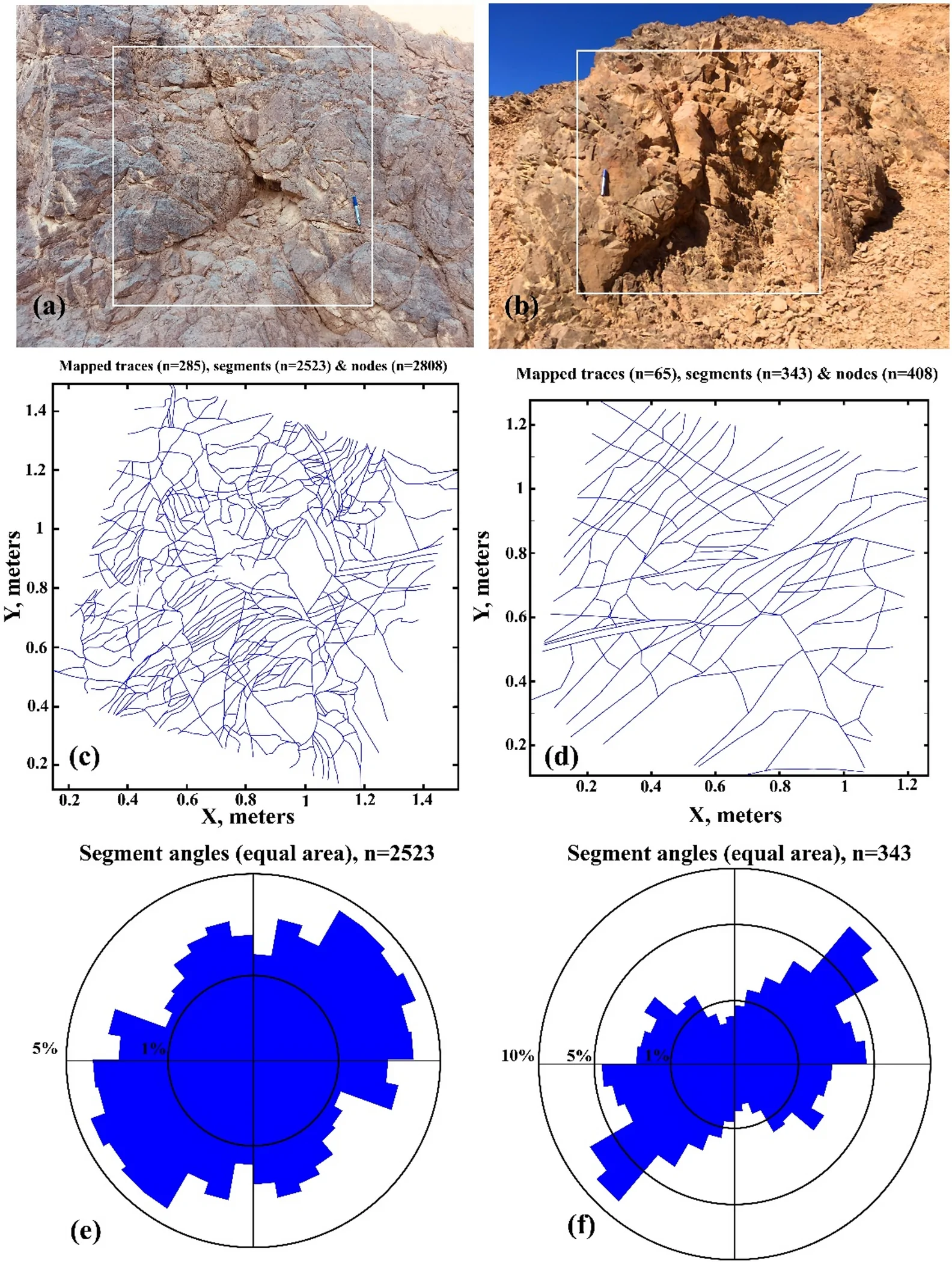
Two fractured networks of younger granite observed in Sid and Atalla locations. The two outcrops of younger granite (**a**) and (**b**) in Atalla in Sid, looking to SSW; (**c**) Fracture trace map of the Sid area shown in (**a**); (**d**) Fracture trace map of the Atalla area shown in (**b**); Angles of fracture segments from a younger granite network and distribution of fracture orientations in the rose diagram (**e**) for the first location and (**f**) for the second location.

Healy et al.^77^ released a MATLAB^®^ script named Fracture Pattern Quantifier (FracPaQ, V. 2.8, 2021) This script aims to serve as a *“software-based toolbox”* for characterizing fracture patterns in 2D. The major applications of the FracPaQ toolbox are the processing of photographic fracture networks and the statistical analysis of the distributions of fracture characteristics and permeability. The open-source FracPaQ software used in this publication quantifies fracture patterns in 2-D from digital outcrop photography. For this paper, fracture trace maps were generated from 2D field images of younger granite in two locations: Sid (Fig. 9a) and Atalla (Fig. 9b). FracPaQ software was used to analyze and evaluate the fracture trace maps of the 2D fracture data. The field photographs were imported into Adobe Illustrator™, where a new layer was created to manually trace the fractures as line elements. Once the digitizing was complete, the networks (Figs. 9c, d) were saved as Scalable Vector Graphics (SVG) files without the underlying original layer. Then, these clean vector files were used for quantifying fracture patterns.

## Results

### Fault kinematic analysis and paleostress reconstruction

**Fig. 10:**
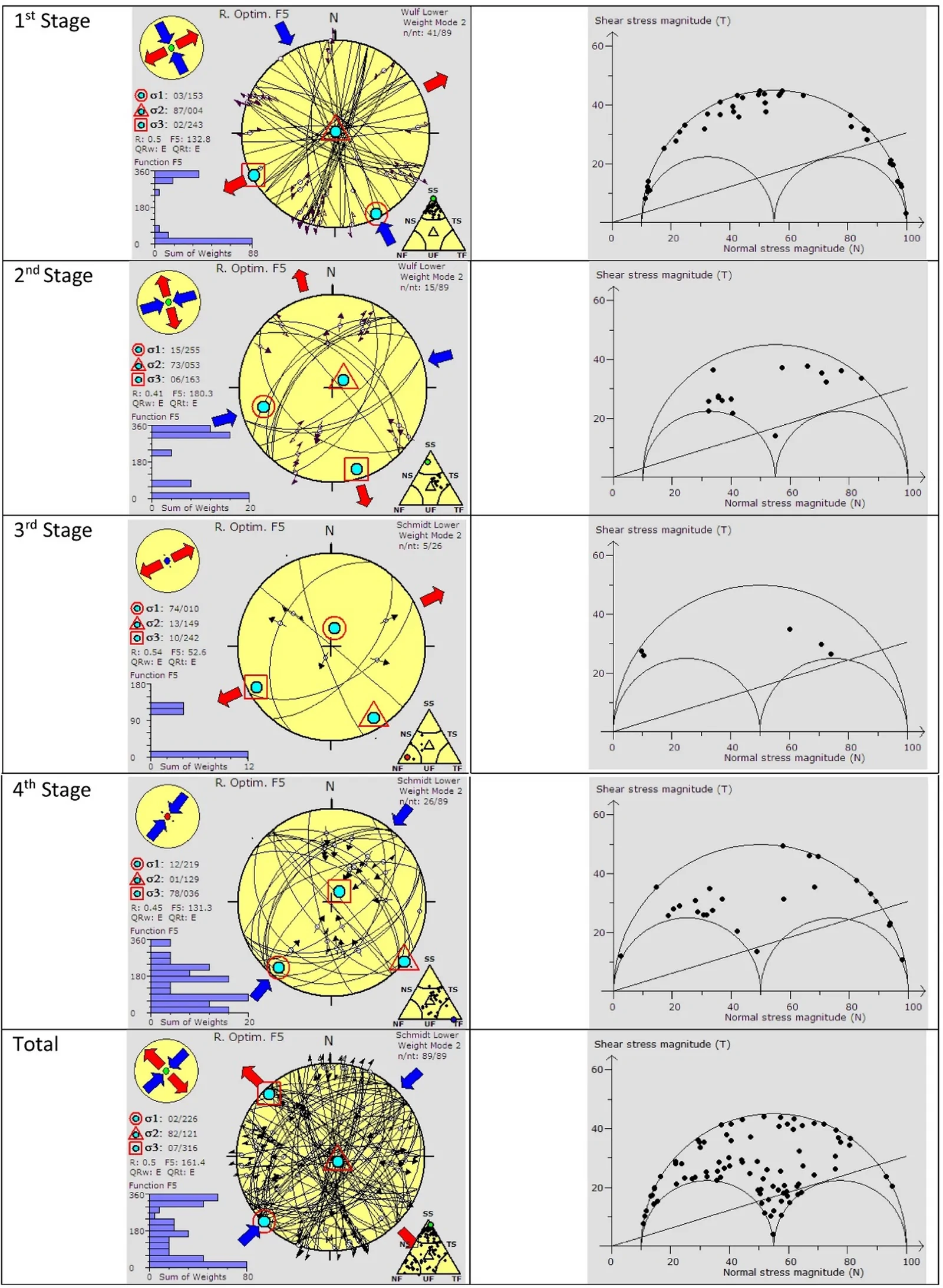
Stress stages 1st‒4th obtained from the processing of all fault data collected from ASSB area. σ1, σ2 and σ3 are the maximum, intermediate and minimum principal stresses, R= stress ratio, n= number of data in a homogeneous subset, nt = total number of data measured, F5 = the misfit function, QRw = quality ranking according to the World Stress Map project, QRt = quality ranking according to the TENSOR scheme. Both QRw and QRt show E (Worst) quality for all the tensors. The stereograms show the fault planes which displayed as great circles and their slickenlines which displayed as white dots with arrows, Mohr circles indicate the relative stress magnitudes on the fault-data, the histograms display the distribution of the values of the misfit function (F5), and the triangular Frohlich diagram represents the type of fault which shown as black dots and the stress regime of the best-fit tensor which shown as green filled dot.

A total of 89 fault surfaces have been measured in Atalla and Sid areas (Table 1). Faults of each area have been processed independently. They have been separated into homogeneous and compatible subsets during the inversion process manually, in an iterative way and with cross-check to verify the fault separation. The relative chronology was established using fault and striae cross-cutting relationships. A total of four successive paleostress states have been identified (Fig. 10).

**Table 1:** Paleostress states (stages) identified from the processing of fault-slip data of Atalla (Area I) and Sid (Area II) and for the two areas together, using Rotational Optimization method and Win_Tensor program.

Area/stage	Method	*n*	nt	S1p1	S1az	S2p1	S2az	S3p1	S3az	*R*	*R′*	*R*′_StD	SH_max_	Sh_min_	SH_StD	Regime
Stage 1: NNW-SSE Transpression																
Area I	Rot. Optim.	15	40	1	339	85	240	5	69	0.5	1.50	0.38	159	75	7.3	SS
Area II	Rot. Optim.	27	49	5	217	85	34	00	127	0.50	1.50	0.37	0.37	127	18.2	SS
Stage 2: ENE-WSW Transtension																
Area I	Rot. Optim.	8	40	2	256	83	00	6	165	0.50	1.50	0.22	75	166	15	SS
Area II	Rot. Optim.	7	49	2	130	81	233	9	40	0.50	1.50	0.2	130	40	16.3	SS
Stage 3: ENE-WSW Extension																
Area I	Rot. Optim.	4	40	63	26	25	182	10	276	0.68	0.50	0.19	008	98	13	NF
Area II	Rot. Optim.															
Stage 4: NE-SW Compression																
Area I	Rot. Optim.	15	40	20	259	2	168	70	73	0.64	2.50	0.25	81	171	31.7	TF
All areas																
Stage 1	Rot. Optim.	41	89	3	153	87	00.4	2	243	0.50	1.50	0.74	153	63	11.7	SS
Stage 2	Rot. Optim.	15	89	15	255	73	53	6	163	0.41	1.50	0.27	74	164	12.8	SS
Stage 3	Rot. Optim.	5	89	74	10	13	149	10	242	0.54	0.50	0.41	154	64	13.8	NF
Stage 4	Rot. Optim.	26	89	12	219	1	129	78	36	0.45	2.5	0.22	39	129	30.4	TF

n: number of fault slip data selected; nt: initial valid data number; R: ratio of principal stress difference; R′: stress regime index; R′ StD: standard deviation 1sigma for R′; SHmax: direction of the horizontal principal stress; Shmin: direction of the horizontal principal stress; SH- StD: standard deviation 1sigma for SHmax.

- Stage 1: transpressional regime with a prolate shape of the stress ellipsoid (R′= 1.5) and NNW-SSE SH_max_ (SH_max_ = N153°E).
- Stage 2: transtensional regime with an oblate shape of the stress ellipsoid (R′= 1.5) and ENE-WSW SH_max_ (SH_max_ = N074°E).
- Stage 3: extension regime (R′= 0.5) and ENE-WSW Sh_min_ (Sh_min_ = N064°E).
- Stage 4: compression regime (R′= 2.5) and NE-WSW SH_max_ (SH_max_ = N039°E).

Among the above mentioned the second shearing stage is the first order control on gold deposit distribution in the ASSB.

### Fracture pattern quantification

#### Orientation plots

The rose plots of the fracture network on younger granite in Sid area confirm a two-abundance orientation of the fracture population in the NE and NW, respectively (Fig. 9e). In the Atalla area, fracture segment angles were plotted: the fracture network with three standard orientations in the NE, E and NW (Fig. 9f).

#### Density (P20) and intensity (P21) maps

**Fig. 11:**
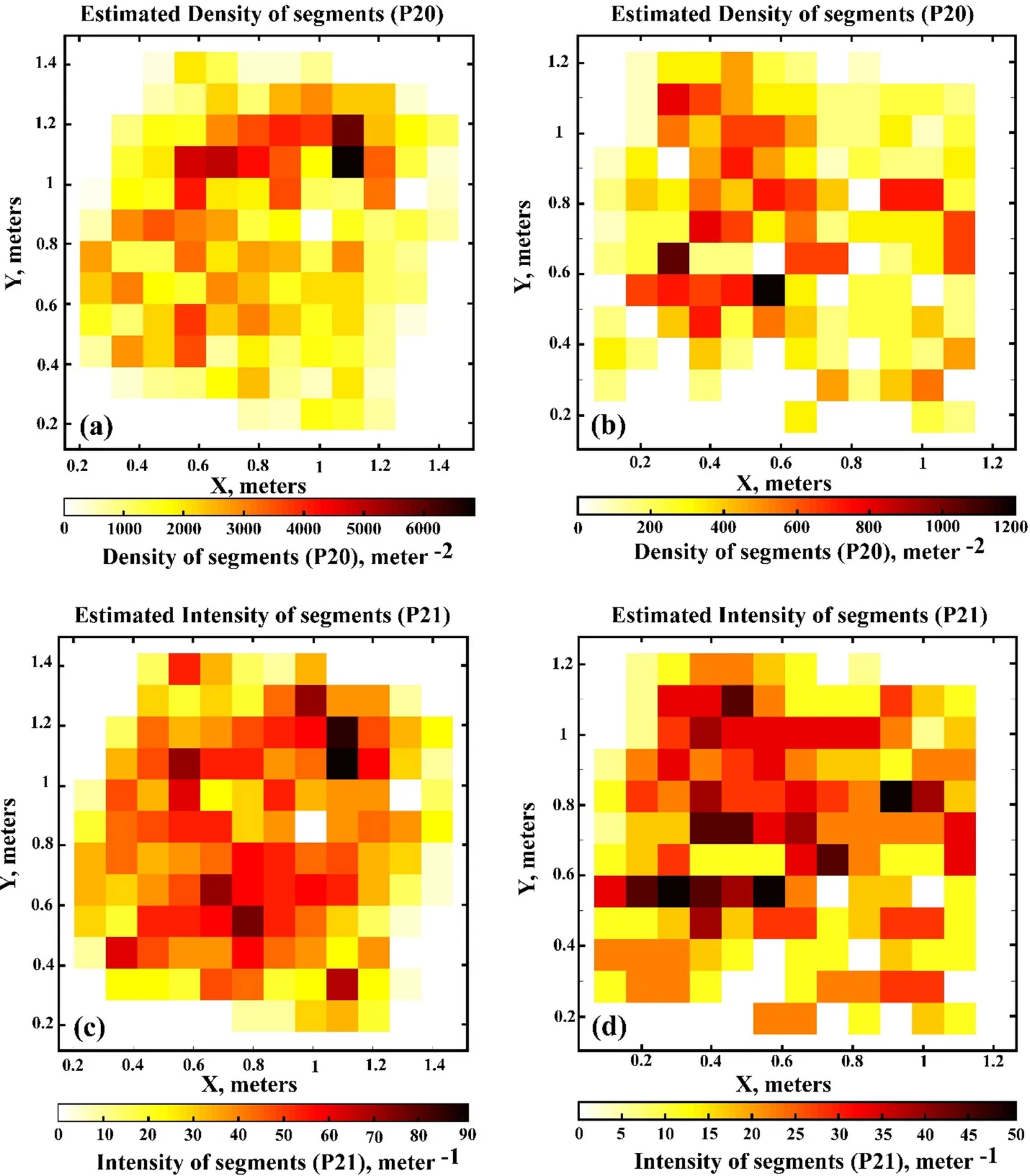
Estimated fracture density maps (P20) and estimated fracture intensity maps (P21). (**a, c**) P20 and P21 maps for fractured younger granite in Sid area; (**b, d**) P20 and P21 maps for Atalla area.

Dershowitz and Herda^82^ designated fracture density as P20, with units of m^− 2^, and described it as the number of fractures per unit area, and fracture intensity as P21, which has units of m^− 1^ and is defined as the sum of all fracture lengths in a given area (thus, m/m^2^ = m^− 1^). The density map of the younger granite fracture network in Sid area (Fig. 11a) reveals one distinct cluster of high density (i.e., many fractures per square metre), with the highest values of density clusters focused in the NE direction. The fracture intensity estimation map (Fig. 11b) exhibits high intensity (considerable fracture lengths per unit area) in two orientations, NE and NW. The density map of the younger granite fracture network in Atalla area (Fig. 11c) reveals two distinct clusters of high density, with the highest density clusters extented from NE-SW to NW. The younger granite fracture intensity map (Fig. 11d) exhibits high intensity extented also from NE - SW to NW.

#### Connectivity and permeability

**Fig. 12:**
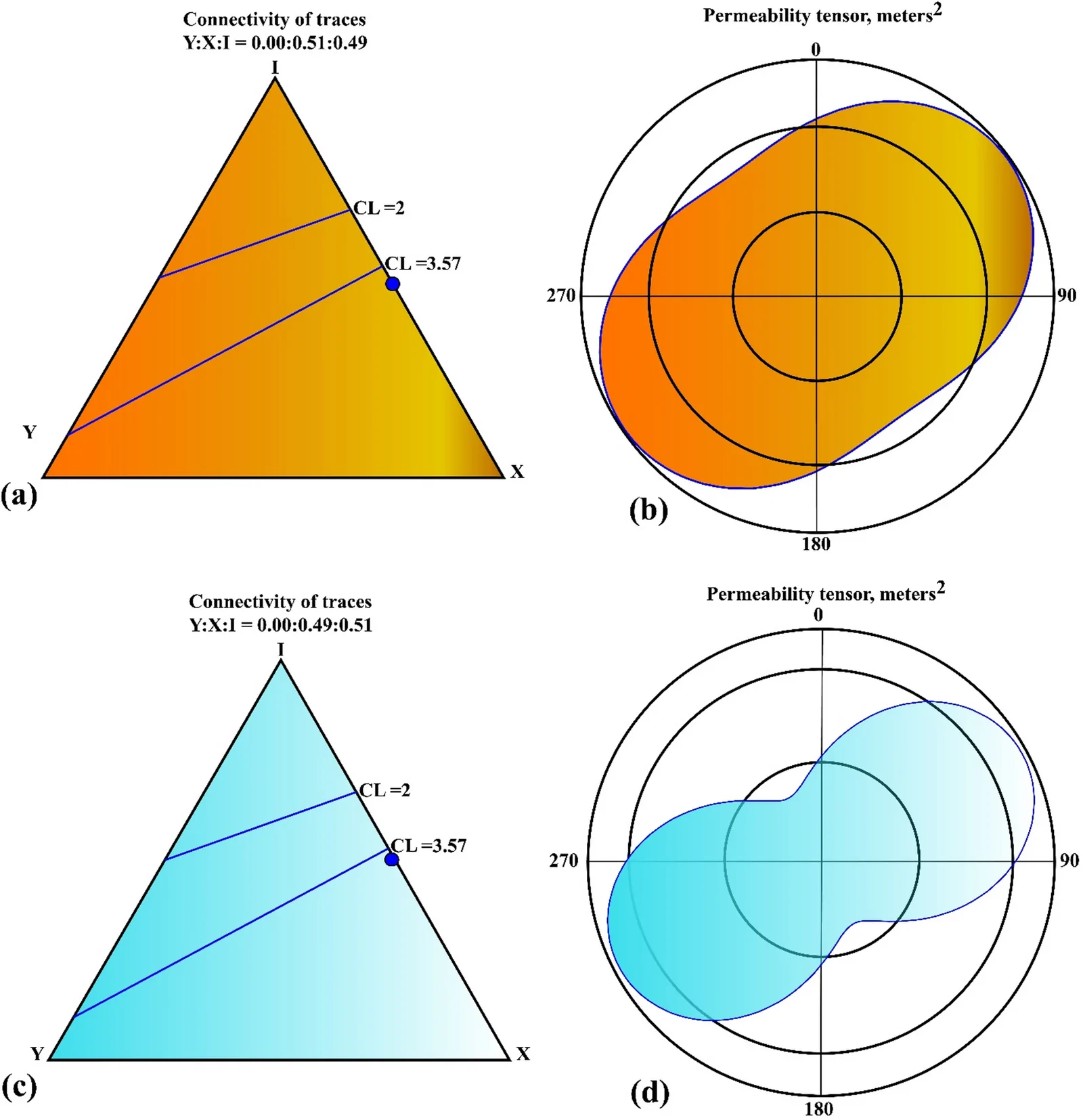
The estimated connectivity and 2D-permeability ellipse of fracture networks. CL refers to the intersections per trace (after^85^). Higher CL values (closed to 3.57) indicate well-connected fracture networks, which are more favorable for fluid flow. (**b**) and (**d**), rose diagrams showing the permeability tensor ellipses. The different concentric circles represent increasing magnitudes of permeability, with directionality indicated by the ellipse shape. The elliptical contours show the anisotropy of permeability and wider lobes indicate directions of enhanced fluid flow.

Recently graph theoiry has been used to explain fracture’s connectivities^83^. The connectivity and the 2D-permeability ellipse plots are linked to research on fluid flow in rock fracture networks^77^. The connectivity diagram depends on nodes as the isolated ends of traces (I), branch points, splays, or abutments (Y), and cross-cutting intersections (X). These nodes are the three vertices of the connectivity ternary plot of the fracture network. In this diagram, more connected networks plot toward the lower Y-X side, while less connected networks plot toward the apex (I)^84^. The term CL-values represents the quantity of connections per trace, with a higher CL value indicating greater connectivity (Fig. 12). Networks that are more interconnected tend to appear towards the bottom of the diagram, as stated by Sanderson and Nixon^85^. In the study area, we studied fracture connectivity and permeability. The connectivity plots (Figs. 12a, c) are similar, with the fracture network of Atalla area having a slightly higher proportion of “Y” nodes, “X” nodes and “I” nodes, reflecting higher connectivity overall. Permeability estimation in Sid area shows that the fracture network in the younger granite (Fig. 12b) parallel to the shorter, high-density fracture set in azimuth 55° (Fig. 12c). For the other location (Atalla area), permeability (Fig. 12d) is oriented parallel to the longer (Fig. 9d), high-density fracture set in azimuth 60°.

## Discussion

### Paleostress history and kinematic framework

The paleostress inversion results for the Atalla-Sid Shear Belt (ASSB) reveal four tectonic evolution stages. These stages display a shift from regional convergence to localized relaxation and subsequent reactivation within the Central Eastern Desert. The oldest stage is characterized by a transpressional regime with a prolate stress ellipsoid (R′=1.5) and NNW-SSE oriented SHmax (N153ºE), where σ2 is vertical and σ1,σ3 are horizontal. This stage aligns with the late Neoproterozoic oblique convergence during the assembly of East and West Gondwana and resulted in the development of the pervasive NW-to-NNW structural grain of the ASSB. Most probably, the major thrust stacks and shear-related folds formed during this phase worked as an initial structural framework for later fluid localization. Stage two showed an oblate stress ellipsoid (R′=1.5) and an ENE-WSW oriented SHmax (N074ºE) which reflect a critical kinematic transition to transtensional regime. This transition is interpreted as a result of crustal relaxation and a change in the far-field stress orientation. The switch to transtension facilitated the opening of NE-to-ENE oriented dilatant zones and shear-related jogs. These structures acted as high permeability conduits that attracted hydrothermal fluids from the dehydrated metamorphic basement. In addition, the oblate shape of the stress ellipsoid during phase suggested a multidirectional strain field that supported the formation of complex, interconnected vein networks instead of single isolated fractures system. The subsequent stage 3 (Extension, R′=0.5) likely reflects the final gravitational collapse of the orogen and stage 4 (NE-SW Compression, R′=2.5) might have caused minor reactivation of earlier faults.

### Granite-hosted fracture networks and fluid pathways

Understanding the structural development of the Atalla-Sid Shear Belt (ASSB) is critical for deciphering the fracture patterns that eventually determined gold mineralization in the region. Paleostress inversion investigations reveal that the initial phase of deformation, Stage 1 NNW-SSE transpressional convergence, established the NW-to-NNW structural grain that would later serve as a framework for hydrothermal fluid localization^67^. While the initial compressional phase established the regional architecture, the transition to Stage 2 had the biggest influence on ore formation. The transition to a transtensional regime, typified by an ENE-WSW directed maximum horizontal stress (SH_max_ = N074°E), appears to have been the critical tectonic event responsible for establishing economically significant mineral routes^86^.

This kinematic change is readily seen in the fracture network data. The high fracture density (P20) and intensity (P21) seen in the Atalla area, which spans orientations from NE-SW to NW, are strongly related to the dilatant zones predicted by Stage 2 transtension^82^. The oblate stress ellipsoid (R′ = 1.5) recovered from paleostress analysis further helps explain why the resulting fracture systems (Figs. 9c–f) are geometrically complex and multi-directional, rather than forming simple planar sets^87^.

Connectivity ternary plots provide the most compelling empirical confirmation of this interpretation: the Atalla network plots consistently toward the Y-X baseline, reflecting a dominance of branching (Y) and cross-cutting (X) nodes over the isolated I-nodes that characterize the comparatively simpler Sid area network^84,85^. This topological contrast shows that tectonic relaxation during Stage 2 allowed the fracture system to exceed a percolation threshold, allowing gold-bearing hydrothermal fluids to rise and circulate efficiently on a vast scale^88^.

When permeability anisotropy is investigated, the relationship between the stress field and fluid flow becomes much more obvious. In the Atalla area, the maximum permeability vector (κmax) trends at roughly 60° azimuth, sub-parallel to both the dominant gold-bearing quartz veins and the Stage 2 SH_max_ orientation. This suggests that the dominant stress field regulated the primary hydraulic conduits in addition to dilating the fractures^89^. Later deformation events, including Stage 3 gravitational collapse and minor Stage 4 compression, likely reactivated some of these structures, but the essential mineralization geometry had already been established during the Stage 2 transtensional episode^90^. These observations confirm that the well-connected fracture topology observed at Atalla expressed through its high proportion of Y and X nodes remains the most reliable structural indicator of efficient fluid transport and focused gold mineralization, in contrast to the more hydraulically restricted conditions that characterize the Sid area.

### Structural controls on gold mineralization of the ASSB

In the ENS (and the entire ANS and EAO), gold deposits occur in a variety of tectonic settings and frequently show spatiotemporal distribution. Gold resources are mainly quartz and/or carbonate veins and veinlets that are injected along specific late Neoproterozoic structural fabric elements, like shears/fractures, stockwork network, brecciated/mylonitized zones, hinges of thrust- and shear zone-related folds, and tectonic contacts (i.e. structurally controlled)^91^. Structural fabrics are regarded to be the main control on gold-bearing quartz and/or carbonate veins. The late timing of OGDs is crucial to geological/structural-based investigation techniques and methodologies. Formation of orogenic gold in the ANS was regarded by many workers^20,^^92-^^94^ to took place just after the end of the arc accretionary stage where the shield became established with a 30–40 km-thick continental crust and experienced a transitional period from compressional-transpressional accretion to post-amalgamation transtensional shearing.

The ASSB is a remarkable high strain zone in the Central Wrench Tectonic Province (Central Eastern Desert) of the ENS^24^^,95^. Most studies in this belt focused on the post-Hammamat felsites (∼28.7 km long by 7.2 km maximum width) that represent one of the main litho-units in ASSB, in terms of petrology and geochemistry and also to their relation to the Um Had granite pluton. All studies have confirmed that the felsites extruded into the mélange rocks and both affected by shearing pre-dating the intrusion of Um Had granite.

**Fig. 13:**
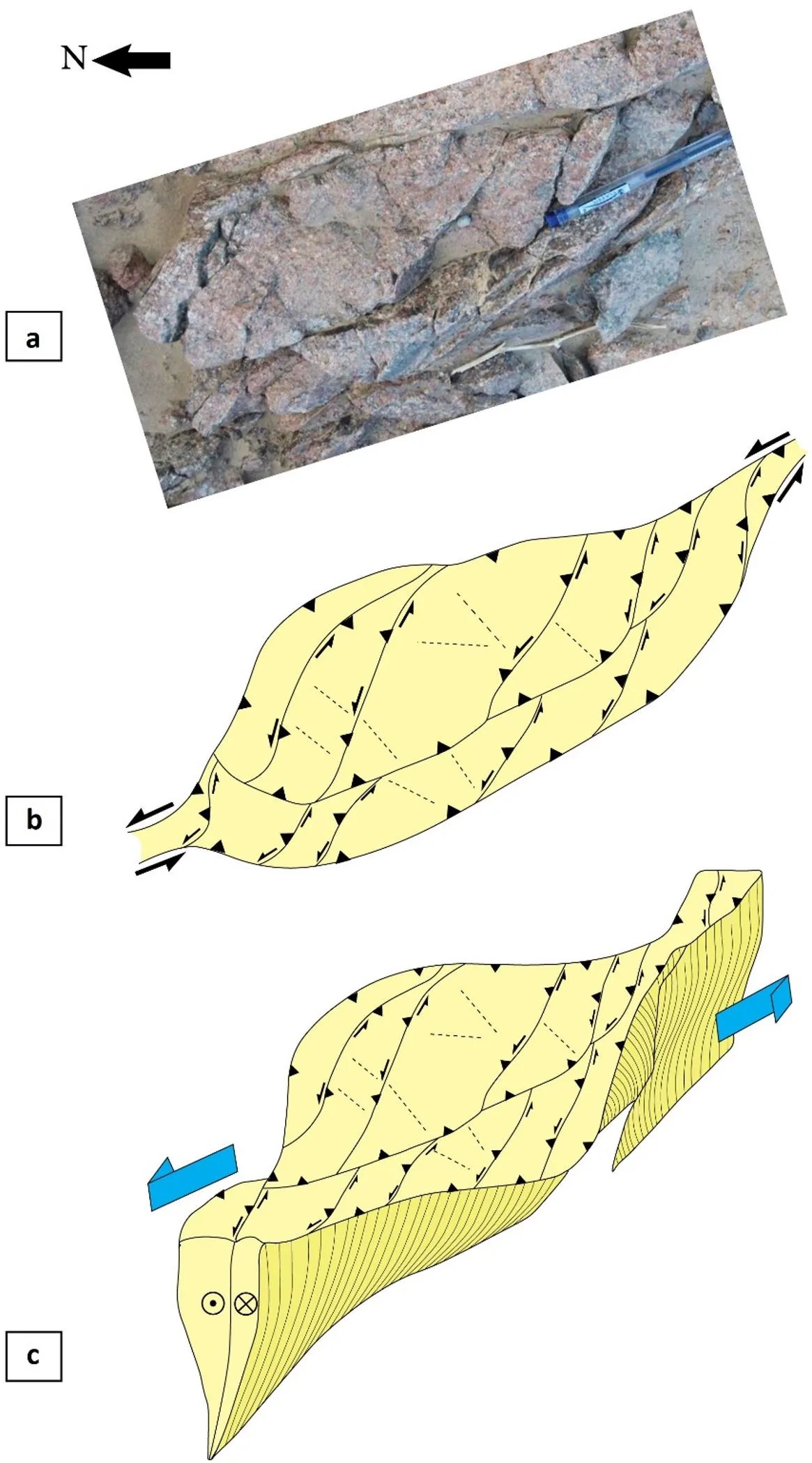
Transpressive structures in Sid area, (**a**) close-up view showing well developed transpressive structures, (**b** and **c**) sketch and 3D block diagram of the same field photo.

The structural pattern of the ASSB was interpreted by Akawy^55^^,56^ as a NE-dipping thrusting with top-to-the SW propagation. Centimeter-to-kilometer scales thrust-related folds with NW-SE axes do exist, and these folds are traversed by four sets of faults; NW-SE (oldest, mainly strike-slip), E-W, N-S and NE-SW (youngest, extensional) (Figs. 2, 3 and 4). It was concluded that a pronounced structural implication in this belt is the transposition of NE-oriented fabric in the Meatiq into NW-oriented pervasive shearing^12^. One important aspect, however, lies in the fact that the transpressive structures in the ASSB (Fig. 13) are attributed to the combination of transcurrent shearing and the ENE-directed shortening, like in many areas in the ANS.

**Fig. 14:**
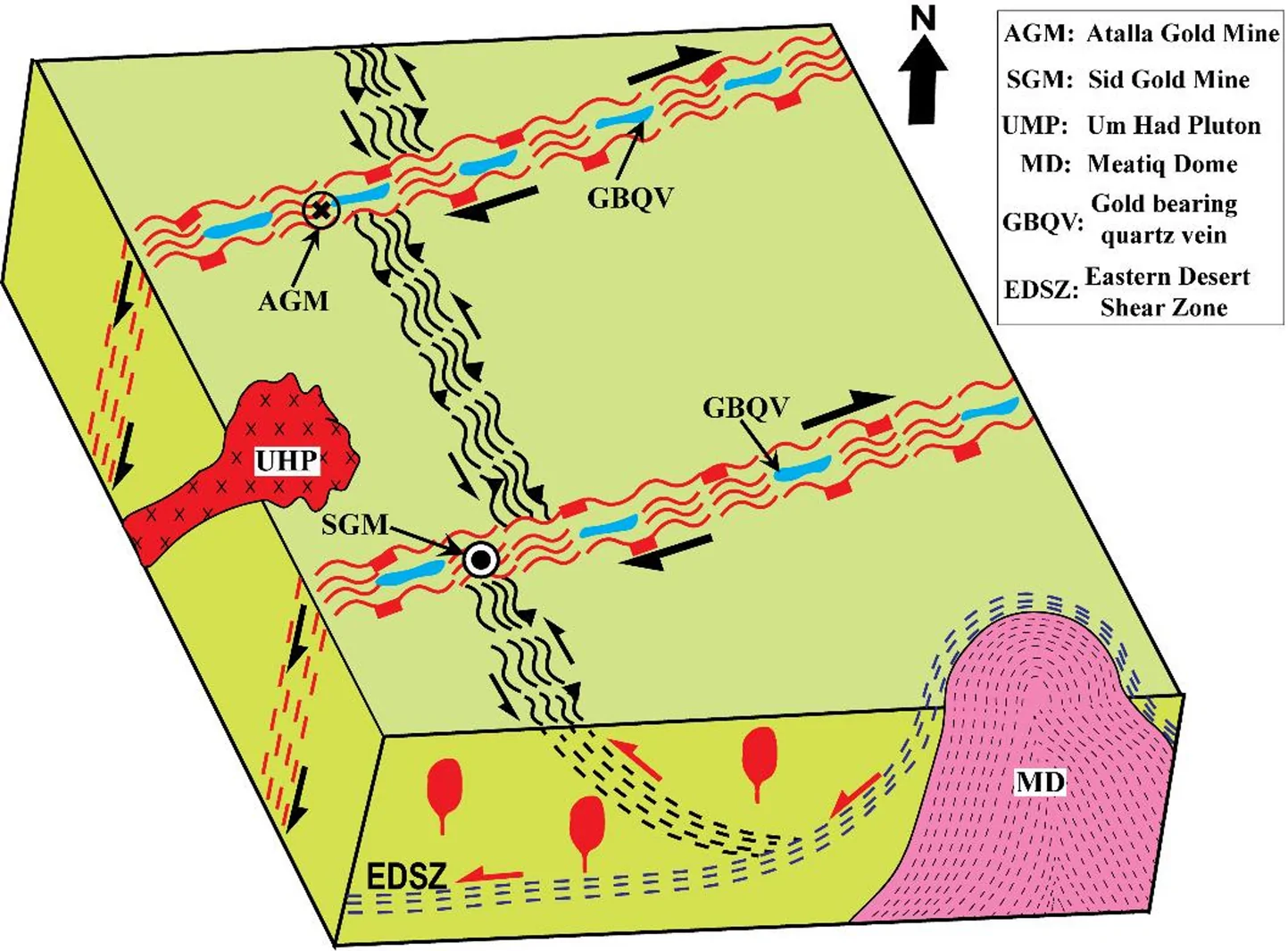
3D-block diagram exhibiting the main shearing trends in the concerned ASSB. The NW- (to NNW-) trend is traversed by the NE- (ENE-) trend with obvious dextral offsetting.

Field examination conducted in the present study revealed that the ASSB squeezed between the Meatiq dome to the east and the Um Had pluton to the west (Fig. 14). It is clear that exhumation of the dome and the emplacement of the pluton have direct impact on the ASSB. The exhumation itself can be attributed to the E-W (to ENE-WSW) oblique convergence between east and west Gondwana lands. As a result, a group of NW- (to NNW-)-oriented thrusts were formed (Fig. 13), as indicated in other areas in the ENS^7,^^9^^,^^12^^,^^96^. The thrusts are frequently exhibiting top-to-the-SW- (to WSW-) vergence and occasionally possessing top-to-the-NE- (to ENE-) propagation.

#### Coi

nciding with the Najd Shear Orogeny, a transition from pure contraction to transpressional regime took place, as in many areas in the Najd Shear Corridor of the ENS (Fig. 13). The NW- (to NNW) transpressional zones can therefore join the oldest deformation stage (Stage 1; R′= 1.5 & SH_max_ = N153°-333°E) in the ASSB (Fig. 10). Significant sinistral sense of shearing can easily be detected by the inspection of transpressive structures and kinematic indicators that show monoclinic symmetry. Another important aspect lies in the fact that the NW- (to NNW-) transpressional zones are dissected by NE- (ENE-) trend with noticeable dextral sense of shearing (Fig. 14). In spite of the overprinting relation between the two shearing trends, some workers^53^^54^, considered both of them as conjugate pairs. Such view can’t be accepted because of the presence of many various scale features which indicate that both trends can’t be formed during the same deformation phase by the same stress regime.

Based on our field investigation, we do believe that the NE- (ENE-) shearing trend formed under semi ductile-semi brittle regime. This shearing trend has a prolonged history of deformation, where this trend suffered a converting from transpression to transtension (Stage 2; R′= 1.5; SH_max_ = N074°-254°E) (Fig. 10); may be due to crustal relaxation and a change in the far-field stress orientation. Parallel to the NE- (ENE) transtensional shearing are dilatant zones and overstep-ping faults that appear to occur during the same stage of deformation (Fig. 14). So, it is particularly significant to carefully investigate such trend of shearing and related dilatant zones. It is critical to realize that these dilational zones (e.g. dilational jogs) are shear zone-related and not related to reactivating earlier/later-formed shears or faults in an opposing far-field stress condition. In the ASSB, this shearing trend acts as conduits for fluid migration from the dehydrated metamorphic zone. In other words, injection of gold-bearing quartz veins along the NE- (ENE-) shears (Figs. 8 and 14) was coeval to this stage. It is of particular importance to denote here that gold-bearing quartz veins and veinlets are infrequently recorded along accommodation faults arrays that tend to be ∼65–70° to the main shearing (Figs. 8 and 14) and so along shear zone- and thrust-related asymmetric overturned antiforms and synforms.

The ASSB underwent two subsequent stages; Stage 3: extension (R′=0.5), ENE-WSW extension (Sh_min_ = N062°-242°E); and Stage 4: (R′= 2.5), NE-SW compression (SH_max_ = N039°-219E°) (Fig. 10). The question that may arise whether the latter two stages played a role in the formation of the OGDs in the ASSB belt. Our field evidence and the lack of gold-bearing quartz veins associated with these stress orientations suggest that these two stages have no controls on gold mineralization. Therefore, they can’t be considered as gold-related deformation, and they may represent the final phases of the Neoproterozoic Orogeny that played a role in the final structural geometry of the belt.

The results obtained from analysis of fault pattern data confirm three-abundance orientations (NW, NE and E) of fracture population in Atalla area, and two orientations (NW and NE) in Sid area. The density map of the younger granite fracture network in Sid area (Fig. 11a) reveals one distinct cluster of NE- and NW- high density clusters in Atalla, and nearly the same clusters in Sid. The permeability estimation of the younger granite fracture network seems to be parallel to the longer high-density fracture set (azimuth 60°) in Atalla, and parallel to the hotter, high-density fracture set (azimuth 55°).

### Role of pre-existing structures in localizing gold mineralization

The ANS was assembled from juvenile crust during a three -stage Neoproterozoic tectonic evolution involving: (1) intra-oceanic subduction and arc accretion stage, (2) orogenic extension stage, and (3) post-extensional compressional stage^97^. Among these stages, stage three played a significant role in localizing gold mineralization in the ENS and geomorphological shaping of the shield rocks. This stage is manifested predominantly by the NW-SE oriented transcurrent Najd shearing and related folding and thrusting, and the E-W (to ENE-WSW) transpressional/transtensional structures, along with the N-S trending shortening zones. The shear and volume strain aspects of Najd shears are described as stress controls on the brittle evolution of Najd faults^97^. The Najd shears and pinnate fracturing are favorable sites, along which the hydrothermal fluids migrate and consequently the base metal vein deposits accumulate.

Such fluids are either of pure metamorphic origin or linked to syn-kinematic granitoids (magmatic-metamorphic model). Injection of fluids led to the formation of complex arrays and network of vein systems. In certain granite-hosted deposits, mineralization is commonly limited to shear zones formed after an intrusion, this could indicate that the physical properties of the solid granite rocks influenced the creation of the structural framework^3^. Fowler^11^ proposed that the presence of structural discontinuities such as domed shear zones and moderately dipping thrusts in the CED aided in the emplacement of calc-alkaline granitoid intrusions, several of which are probably linked to hydrothermal processes and the formation of gold deposits. According to Helmy & Zoheir^98^, the Fawakheir intrusion is believed to have supplied the necessary fluid and heat to support the hydrothermal system, while the serpentinite country rocks act as a source of metals for the Sid gold deposit. While Atalla gold deposit in the CED is situated within the Atalla granitic intrusion, adjacent to a significant NNW-trending left-lateral shear zone known as the Atalla shear zone^99,100^.

The presence of late tectonic granitoid intrusions within the same shear zone suggests that both the Atalla and Sid gold deposits share similar genetic origins tied to tectonic and/or magmatic events around 600 million years ago^2,100,101^. Some workers^102^ believed that the fluids were aqueous-carbonic, generated at a greenschist/amphibolite transitional facies. Other authors^94^ attributed gold mineralization to the period between the regional transpression and transtension stages.

### Geodynamic implications of Stage 2 (transtension) to Stage 1 (transpression)

Many workers reported a long-lasting kinematic history of the Najd-related NW trending left-lateral transcurrent shearing and NE- (to ENE-) trending right-lateral shearing. There is nearly a total agreement that both trends formed under a transpressional regime, making both as typical examples of transpressional zones in the worldwide continental shields. However, the relation between both shearing trends remains a matter of dimensions and a great debate. For example, there are two principal schools of thought regarding the kinematics of both trends. One group of researchers^103-^^105^ reported that the kinematic history of the NW-shearing switched from dextral at the beginning phase to sinistral movement. Another group^50^supposed that the prevailing kinematic history was sinistral and continued with the same sense. In the present work, we evidently show that the NE- (to ENE-) shearing trend was transpression at the beginning and the tectonic regime switched to transtensional one. In all cases, although some workers^53,106^, have been thought that the NW- and the NE- (to ENE-) shearing trends to be conjugates, in the present study and based on the overprinting relations we do believe that the NE- (to ENE-) trend is younger than the NW-trend. Apart from the concerned shear belt, it is obvious that both trends play a role in controlling gold mineralization in the ENS (and the whole ANS).

## Conclusions

Based on our obtained results, and data interpretation and analysis, the following concluding remarks can be drawn:

- Two main shearing trends are encountered in ASSB.
- The first shearing trend has NW- to NNW- and shows evidence for transpressional regime, documented by top-to-the-SW- to WSW- thrusts with occasional top-to-the-NE- (to ENE-) vergence. It is affiliated to an oldest deformation stage (Stage 1; R′= 1.5 & SH_max_ = N153°-333°E) in the ASSB.
- The NW- to NNW-shearing trend (the first-order trend) is a belt-parallel pervasive structural trend.
- The second shearing trend follows the NE- to ENE- direction. It is evidently transtensional and younger with obvious dextral offsetting. It is akin to Stage 2: transtensional regime with an oblate shape of the stress ellipsoid (R′= 1.5) and ENE-WSW SH_max_ (SH_max_ = N074°E).
- Shearing along the second converted from transpression to transtension, mostly due to far-field condition or crustal relaxation.
- The NE- to ENE-oriented transtensional shearing was clearly pre-date gold mineralization in ASSB.
- As in the majority of world-class OGDs, the OGDs in the ASSB are remarkably late in the kinematic history of its hosting belt.
- The structural geometry in Sid is more complicated because of the presence of granitic intrusion that impose on more ductile metaultramafic envelope.
- Obtained results from the present study should be considered in future gold exploration strategies in the ENS and the ANS, as well as in similar worldwide orogens.
- It is recommended to carry out geochronological studies on gold-bearing shear belts to constrain timing of mineralization relative to the deformation stages.

## Data Availability

The datasets used and/or analyzed during the current study are available from the corresponding author upon reasonable request.
